# Forty-nine-month survival in a metastatic renal cell carcinoma patient across six lines of targeted therapy

**DOI:** 10.3332/ecancer.2014.406

**Published:** 2014-02-25

**Authors:** T Raja

**Affiliations:** Apollo Hospitals, 320, Mount Road, Chennai 600 035, India

**Keywords:** metastatic renal cell carcinoma, everolimus, sequential therapy

## Abstract

A better understanding of the aetiopathogenetic molecular targets in renal cell carcinoma (RCC) and the subsequent advent of targeted therapeutic agents have greatly improved the management and prognosis of RCC and patient survival. However, optimising therapeutic outcomes through appropriate sequential or combination therapy remains a challenge. Our 45-year-old male patient presented with metastatic renal cell carcinoma (mRCC); we effectively managed his aggressive, progressive disease across six lines of treatment, including sequential monotherapy and combination therapy, with targeted agents like sunitinib, everolimus, sorafenib, temsirolimus, and bevacizumab, resulting in a >48-month survival. Appropriate therapy with agents that have non-overlapping target profiles minimised treatment-related toxicities, enabling our patient to tolerate therapy at full doses. This case represents a good example of a significant clinical benefit of targeted therapy beyond the fourth line in mRCC. The survival and prognosis of mRCC patients may thus be significantly improved with the suitable use of newer targeted agents.

## Introduction

Renal cell carcinoma (RCC) has been one of the most widely studied cancers in recent years; however, despite a considerable improvement in our understanding of RCC tumour biology, the unpredictable and aggressive natural history of RCC and the associated complexities in its management often pose important clinical dilemmas. Almost one third of RCC patients present with metastatic disease, and half of those who undergo surgery eventually develop distant metastasis [1–3]. The relatively low response rates and significant toxicities of cytokine therapy, the only available treatment for metastatic renal cell carcinoma (mRCC) until recently, have spurred extensive research, leading to the approval of many targeted agents for mRCC, including vascular endothelial growth factor (VEGF)-directed therapies (bevacizumab, sunitinib, sorafenib, pazopanib, and axitinib) and mammalian target of rapamycin (mTOR) inhibitors (everolimus and temsirolimus) [4, 5]. Studies aiming to determine the optimal use of these agents, including the most effective sequence of therapy and the relative efficacy of combination versus sequential single-agent therapy, are ongoing; however, presently, physician preferences, drug toxicity profiles, patient compliance, and therapy costs usually dictate the choice of targeted therapy. Here, we describe a case of mRCC with lung metastasis at presentation that was treated effectively for four years with six lines of targeted agents sequentially and in combination.

## Case report

A 45-year-old male presented to our centre in October 2008 for medical management of mRCC; his personal and family history was insignificant, and he had no major co-morbidities. In May 2008, he had been diagnosed with advanced RCC (left kidney) that had metastasised to the lungs along with tumour thrombi in the left renal vein. He underwent a left radical nephrectomy with regional lymphadenectomy in the same month, and surgical histopathology revealed grade II clear cell RCC.

Post nephrectomy, the patient was prescribed sunitinib (50 mg/day orally) from September 2008, and he received a total of six cycles of sunitinib until May 2009. Subsequently, the patient remained disease free for five months, and a follow-up PET-CT in October 2009 revealed local recurrence of the tumour in the left renal fossa (LRF) along with mediastinal, retroperitoneal, bilateral hilar and left supraclavicular nodal and bilateral lung metastases, indicating disease progression. The patient was then prescribed everolimus (10 mg/day orally). The patient continued therapy for six months and was tolerating the drug well. Then, an early post-treatment follow-up PET-CT conducted in May 2010 showed an increase in the size and metabolic activity of the soft tissue thickening in the LRF and a 10–15% increase in the number, size, and metabolic activity of the retroperitoneal, mediastinal, bilateral hilar and left supraclavicular lymph nodal and bilateral lung metastases, indicating progressive disease.

**Figure 1.**
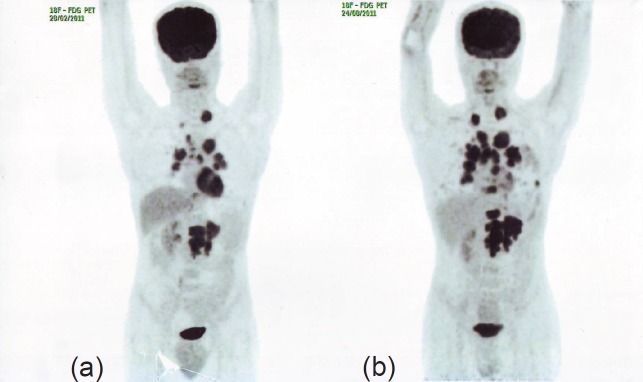
PET-CT in August 2011 (b) shows ~35–40% increase in the size of the LRF mass and appearance of multiple vertebral lesions compared to PET-CT in February 2011 (a), indicating disease progression.

After the failure of two lines of targeted therapy, the patient was advised salvage chemotherapy with sorafenib (800 mg/day orally in two equal doses) from May 2010; the patient continued therapy until a follow-up PET-CT conducted six months later revealed a ~20% increase in the size and metabolic activity of the mediastinal lymph nodal and bilateral pulmonary metastases and the appearance of a new metabolically active metastatic focus in the upper lumbar vertebral body, indicating disease progression. Considering the apparent inadequacy of disease control by monotherapy, the patient was advised salvage immunochemotherapy with a combination of bevacizumab (400 mg IV every two weeks), vinblastine (0.11 mg/kg IV bolus injection on days 1 and 2 every 21 days), and mitomycin C (12 mg/m2 IV every seven weeks) from November 2010. The patient continued therapy for eight cycles until February 2011; however, a routine follow-up PET-CT, six months after discontinuation of therapy, showed a ~35–40% increase in the size of the LRF mass and appearance of multiple vertebral lesions, indicating skeletal metastases, and hence, further disease progression (Figure 1). The ECOG status improved from 2 to 1 with the first four lines of therapy; however, the ECOG status increased to 2 with increased vertebral and pulmonary lesions.

**Figure 2.**
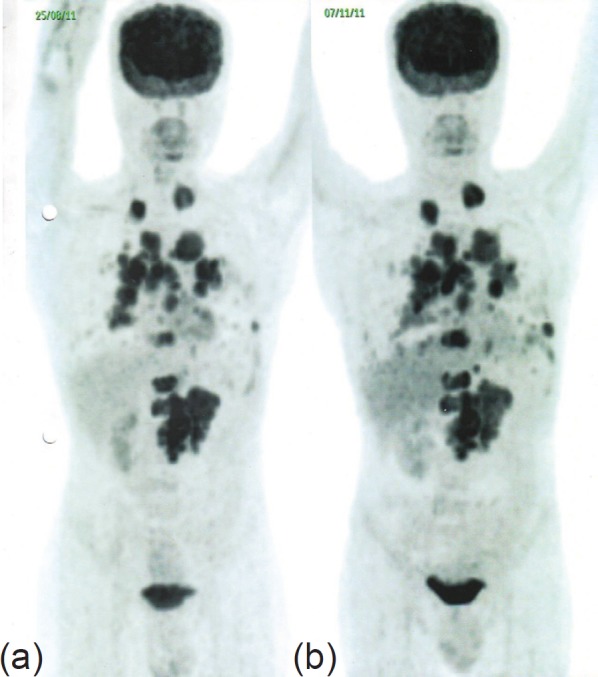
PET-CT in November 2011 (b) shows ~50–100% increase in the size of the pulmonary lesions, ~15% increase in the size of the LRF mass, and appearance of new pleural deposits compared to PET-CT in August 2011 (a), indicating disease progression.

The patient was then prescribed single-agent palliative chemotherapy with temsirolimus (25 mg IV weekly); however, his response over eight weeks was clinically sub-optimal, and an early post-treatment follow-up PET-CT revealed a ~50–100% increase in the size of the pulmonary lesions, a ~15% increase in the size of the LRF mass, and appearance of new pleural deposits, indicating further disease progression (Figure 2).

The patient was therefore prescribed a third salvage therapy with a combination of bevacizumab (400 mg IV every two weeks) and everolimus (10 mg/day orally) along with concurrent radiotherapy for metastatic disease in the bone.

**Figure 3.**
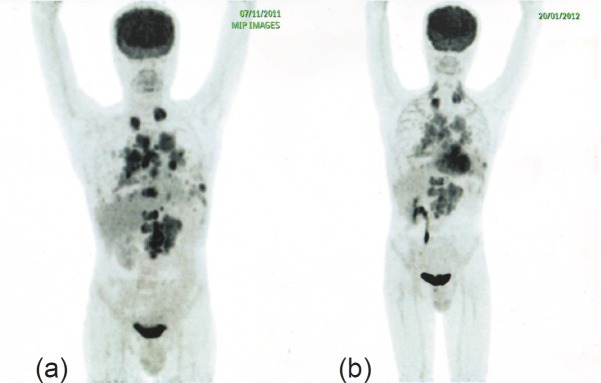
PET-CT after initiation of therapy with everolimus and bevacizumab (b) shows a considerable reduction in the size and metabolic activity of the LRF mass, prevascular, right hilar, and retrocrural lymph nodal, pulmonary, and skeletal metastases compared to PET-CT before therapy.

A routine follow-up PET-CT within two months of initiation of therapy with everolimus and Bevacizumab showed a considerable reduction in the size and metabolic activity of the LRF mass, prevascular, right hilar, and retrocrural lymph nodal, pulmonary, and skeletal metastases, indicating a favourable response to therapy with a significant clinical benefit (Figure 3). Thus far, the patient has completed seven cycles of treatment with this combination, and the eighth cycle is in progress.

Recently conducted routine follow-up investigations, including a pulmonologist review, were insignificant, except mild pericardial effusion anterior to the right atrium on echocardiography. The patient appears to be tolerating the drugs well, with occasional stomatitis, diarrohea, and fatigue as therapy-related adverse events (TRAEs), and continues concurrent radiotherapy along with adequate nutritional supplementation and supportive care.

## Discussion

Although surgery remains the mainstay of RCC treatment, metastatic disease warrants medical management [6]. The erstwhile relative dearth of treatment options for mRCC has been transformed into an embarrassment of the riches [5], owing to our much improved understanding of the various aetiopathogenetic signal transduction pathways and targets in mRCC. As many as seven new agents for mRCC have been approved in less than a decade [7]. However, a therapy with these targeted agents is rarely curative, and therapeutic resistance is common; therefore, patients often have to rely on multiple lines of therapy for a sustained clinical benefit [8]. Many ongoing studies are hence focused on determining the most optimal sequence of these targeted therapeutic agents and the relative efficacy of sequential monotherapy versus combination therapy.

In the first-line (treatment-naïve) setting, national comprehensive cancer network (NCCN) guidelines offer category 1 recommendations to sunitinib, pazopanib, bevacizumab + IFN-α (all for favourable-or intermediate-risk mRCC), and temsirolimus (the only agent with a significant overall survival benefit in poor-risk mRCC), based on data from at least one phase III study for each agent [5]. Interleukin and sorafenib are the other available options for these patients. Sunitinib was shown to be associated with a median progression-free survival (PFS) of 11 months versus IFN-α (median PFS, five months; HR: 0.821, 95% CI: 0.673–1.001, *P* = .051) in a large phase III trial of 750 treatment-naïve patients with mRCC, which led to its approval [9]. Our patient was also advised sunitinib (50 mg/day orally) after surgery.

Although targeted agents have demonstrable anti-tumour activity and prolonged PFS in mRCC, patients often develop resistance to first-line VEGFR-TKI therapy within 6–11 months of therapy and eventually experience disease progression [10]. Preclinical data suggest some mechanisms for the development of VEGFR-TKI resistance, including reemergence of tumour vasculature or upregulation of alternate tumour survival and invasiveness pathways. Consequently, both sequential monotherapy and combination therapy with targeted agents are being explored, since they can potentially diminish the impact of tumour angiogenic escape mechanisms and prolong disease control.

Clinical practice guidelines in the United States and Europe uniformly recommend everolimus as the standard of care in mRCC that has failed first-line VEGFR-TKI therapy, based on robust clinical evidence from the 410-patient RECORD-1 study [11]. Our patient, therefore, was advised therapy with everolimus (10 mg/day orally) after sunitinib failure. There are, however, previous and current, retrospective and prospective, studies on the clinical application of a second VEGFR-TKI (such as axitinib) in first-line VEGFR-TKI failure, based on its relative potency and target selectivity [10]. Sequential administration of VEGFR-TKIs may result in cumulative class-effect toxicities, such as hypertension, hand–foot syndrome, and rash, owing to overlaps between their target selectivity. Since the toxicity profiles of mTOR inhibitors and VEGFR-TKIs do not overlap, the use of a second-line mTOR inhibitor following first-line VEGFR-TKI failure minimises the chances of VEGFR-TKI-associated class-effect toxicities. However, definitive guidance on the relative efficacy of a second VEGFR-TKI versus an mTOR inhibitor in first-line VEGFR-TKI failure appears to be lacking and can be derived only from large, randomised head-to-head comparisons of these agents. Until such data become available, the choice of second-line therapy after first-line VEGFR-TKI failure would be determined by careful consideration of factors such as the distinct safety profiles of targeted agents, patient history, and co-morbidities.

In the case of mRCC progression beyond second-line therapy, currently, there exists no level 1 evidence for any targeted agent, although dovitinib is being studied in a phase III trial in this setting [12]. However, in clinical practice, reintroduction of a VEGFR-TKI following disease progression on a VEGFR-TKI and an mTOR inhibitor is increasingly being applied, based on sparsely available data [4]. VEGFR-TKI rechallenge may be associated with a sub-optimal clinical benefit owing to partial cross-resistance. Our patient was also advised a VEGFR-TKI (sorafenib) after disease progression on everolimus; however, he experienced subsequent disease progression on therapy. Therapeutic agents that are capable of inhibition of multiple angiogenic pathways over a wide spectrum, in addition to VEGF signalling, are therefore being evaluated in the third line.

Although renal cancer is largely resistant to chemotherapy, with response rates of 5–15%, some chemotherapeutic agents, such as 5-fluorouracil, have been shown to have some activity in RCC [13]. Therefore, chemotherapy is generally used in combination with other therapies or can be a therapeutic option for patients who have failed to respond to targeted therapy. Clinical evidence of a fourth line of therapy in mRCC was recently reported in a 52-year-old male patient, treated sequentially with sunitinib, everolimus, sorafenib, and temsirolimus, leading to a PFS of 48 months; therapy with each agent was well tolerated, and there was no apparent cumulative toxicity, suggesting that patients could continue to derive clinical benefit from multiple lines of therapy [14]. Resistance to both mTOR- and VEGF-directed therapies appears to be at least partially transient; thus resensitisation might be an option in patients who exhibit good tolerability to treatment, allowing sustained disease control through multiple iterations of therapy.

Combination therapy is alternatively being evaluated in mRCC, with the goal of achieving additive or synergistic anti-tumour effects, including enhanced tumour shrinkage or a more durable response; however, concerns regarding cumulative toxicities of these agents due to target profile overlaps remain [15]. Although the rationale for combined inhibition of critical pathways remains strong, attempts at combining the currently available targeted agents have been discouraging. Some combinations, notably bevacizumab/sunitinib, have been unexpectedly toxic, whereas bevacizumab/sorafenib warranted the use of reduced dosages of both agents. A recent phase I study of bevacizumab/sunitinib reported an ORR of 52%, higher than that expected with either agent alone; however, the combination resulted in an increased frequency of grade 3 or 4 hypertension, proteinuria, and thrombocytopaenia. Similar results were obtained in another phase I trial combining bevacizumab/sorafenib [16]. Since these studies involved a combination of two VEGF-targeted agents, the amplification of treatment-related toxicities offset the observed enhancement in tumour response to therapy.

The biological hypothesis surrounding a synergistic effect of inhibiting both VEGF and mTOR signalling with specifically targeted agents is compelling [17]. Two phase II studies evaluating combined VEGF and mTOR inhibition (bevacizumab/everolimus; bevacizumab/temsirolimus versus sunitinib versus bevacizumab/interferon) have been reported. A phase III CALGB trial is currently investigating second-line use of everolimus alone versus a combination of everolimus and bevacizumab [16]. Both these agents are relatively specific inhibitors and, in contrast to other combinations of targeted agents, can be administered concurrently at full doses [18].

The advent of several targeted agents and ongoing research on many more are anticipated to significantly improve the prognosis of mRCC. However, in the future, the clinician’s priority would be the optimal identification and application of these agents to balance clinical benefit with the quality of life of the patient. Ongoing studies on sequential and combination therapy with targeted agents in mRCC are expected to provide insights into the most optimal management approaches and further enhance survival.

## Conclusion

This patient represents a case of effective medical management of clinically aggressive, metastatic RCC through the application of sequential and combination therapy with VEGF- and mTOR-directed agents over six lines, resulting in a survival of more than 48 months. Appropriate sequential monotherapy and combination therapy with agents that have non-overlapping target profiles minimised treatment-related toxicities, thereby enabling our patient to tolerate therapy at full doses. Survival and prognosis of mRCC patients can thus be significantly improved with suitable use of newer targeted therapeutic agents.

## Conflicts of interest

The author has no conflicts of interest to declare.

## References

[1] Bukowski RM, Negrier S, Elson P (2004). Prognostic factors in patients with advanced renal cell carcinoma: development of an international kidney cancer working group. Clin Cancer Res.

[2] Belldegrun AS (2007). Renal cell carcinoma: prognostic factors and patient selection. Eur Urol Suppl.

[3] Ljungberg B (2004). Prognostic factors in renal cell carcinoma. Scand J Surg.

[4] Oudard S, Elaidi RT (2012). Sequential therapy with targeted agents in patients with advanced renal cell carcinoma: optimizing patient benefit. Cancer Treat Rev.

[5] Pal SK, Vogelzang NJ (2011). Managing refractory metastatic renal cell carcinoma: a RECORD spinning on a tilted AXIS. Clin Genitourin Cancer.

[6] Rini BI, Rathmell WK, Godley P (2008). Renal cell carcinoma. Curr Opin Oncol.

[7] Motzer RJ (2011). New perspectives on the treatment of metastatic renal cell carcinoma: an introduction and historical overview. Oncologist.

[8] Bracarda S (2012). Everolimus in metastatic renal cell carcinoma patients intolerant to previous VEGFR-TKI therapy: a RECORD-1 subgroup analysis. Br J Cancer.

[9] Motzer RJ (2007). Sunitinib versus interferon alfa in metastatic renal-cell carcinoma. N Engl J Med.

[10] Oudard S, Ravaud A, Escudier B (2010). Sequencing of therapeutic agents in the treatment of advanced renal cell carcinoma: focus on mechanism of action. Ann Urol.

[11] Motzer RJ (2010). Phase 3 trial of everolimus for metastatic renal cell carcinoma: final results and analysis of prognostic factors. Cancer.

[12] Motzer RJ (2012). Phase III trial of dovitinib (TKI258) versus sorafenib in patients with metastatic renal cell carcinoma after failure of anti-angiogenic (VEGF-targeted and mTOR inhibitor) therapies. J Clin Oncol.

[13] Mulders PF (2008). Guideline renal cell carcinoma.

[14] Oudard S (2010). More than 4 years of progression-free survival in a patient with metastatic renal cell carcinoma treated sequentially with sunitinib, everolimus, sorafenib, and temsirolimus. Anticancer Res.

[15] Escudier B (2009). Sequential therapy in renal cell carcinoma. Cancer.

[16] Heng DYC, Choueiri TK (2012). The evolving landscape of metastatic renal cell carcinoma. ASCO.

[17] Stadler WM (2010). Bevacizumab and everolimus in renal cancer: a rational way forward. J Clin Oncol.

[18] Hainsworth JD (2010). Phase II trial of bevacizumab and everolimus in patients with advanced renal cell carcinoma. J Clin Oncol.

